# Comparison of portal vein hemodynamics with ultrasound-based elastography for the prediction of liver fibrosis in patients with chronic liver disease

**DOI:** 10.1038/s41598-023-30279-7

**Published:** 2023-02-28

**Authors:** Kanji Yamaguchi, Yuya Seko, Takamitsu Sakai, Satomi Kitano, Hiromi Okabe, Seita Kataoka, Michihisa Moriguchi, Atsushi Umemura, Yoshito Itoh

**Affiliations:** 1 Molecular Gastroenterology and Hepatology, Graduate School of Medical Science, Kyoto Prefectural University of Medicine, 465 Kajii-Cho, Kawaramachi-Hirokoji, Kamigyou-Ku, Kyoto, 602-8566 Japan; 2 Department of Clinical Laboratory, University Hospital, Kyoto Prefectural University of Medicine, Kyoto, Japan; 3 Department of Pharmacology, Kyoto Prefectural University of Medicine, Kyoto, Japan

**Keywords:** Hepatic portal vein, Liver fibrosis

## Abstract

Chronic liver disease includes nonalcoholic fatty liver disease, progresses from steatosis and hepatitis to fibrosis and cirrhosis, with hemodynamic changes in portal blood flow. This study aimed to compare portal vein hemodynamics with liver stiffness (LS) and steatosis and included 28 subjects with chronic liver disease, in whom LS and steatosis were evaluated in the same image employing two elastography techniques: transient elastography (TE) with controlled attenuation parameter (CAP) using a FibroScan and two-dimensional shear-wave elastography (2D-SWE) with attenuation imaging (ATI). Additionally, peak maximum velocity (V_max_) of the right portal vein and spleen stiffness with 2D-SWE were evaluated. A strong positive correlation was present between LS values obtained with TE and 2D-SWE and between the attenuation coefficients of steatosis obtained with CAP and ATI. Additionally, a negative correlation was present between LS values and the V_max_ of the right portal vein (r = 0.415, p = 0.031). The optimal V_max_ cutoff value for discriminating liver fibrosis with an LS value of > 5 kPa was < 17 cm/s; the ability of V_max_ to predict fibrosis was comparable to that of the FIB4-index. Low V_max_ of the right portal vein was useful for identifying liver fibrosis in patients with chronic liver disease.

## Introduction

While recent advances have allowed the cure and effective control of viral hepatitis, obesity and diabetes are on the rise, with a parallel and rapid increase in fatty liver in patients with metabolic syndrome^1,2^. Metabolic syndrome is a group of conditions that together increase the risk of coronary heart disease, diabetes, stroke, and other serious health conditions. Metabolic dysfunction-associated fatty liver disease, formerly termed as nonalcoholic fatty liver disease (NAFLD), which is considered as a more appropriate term to describe liver disease associated with metabolic dysfunction, affects about a quarter of the world’s adult population and imposes a significant health and economic burden in all societies^3,4^.

Imaging modalities such as ultrasound, computed tomography, and magnetic resonance imaging are often used for the diagnosis of chronic liver diseases including viral hepatitis and NAFLD. In addition, hepatic fibrosis and steatosis are utilized as predictors of advanced liver disease and hepatocellular carcinoma. Elastography, which is a noninvasive method to detect the mechanical properties of tissues, renders liver biopsy almost unnecessary^5,6^. There are four main elastography methods: transient elastography (TE), point shear-wave elastography, two-dimensional elastic wave elastography (2D-SWE), and magnetic resonance elastography^7^. In recent years, many centers have focused on the utility of splenic elastography for the evaluation of the liver fibrosis stage using the extent of spleen stiffness in patients with liver disease and for the assessment of clinical outcomes of liver cirrhosis and its complications, especially the degree of portal hypertension^8–10^. On the other hand, Doppler ultrasonography is used for the noninvasive hemodynamic assessment of hepatic vascular flow in patients with liver diseases^11–13^. Doppler ultrasonography can be easily used in medical practice. Previous studies demonstrated hepatic blood flow generally decreases with disease progression in chronic liver disease^14–17^. Numerous studies reported the correlation between hepatic blood flow and the severity of liver disease, especially in patients with liver cirrhosis^18^. Recent studies have provided important findings on the relationship between the degree of fat accumulation and hemodynamics of the hepatic artery, hepatic vein, and portal vein^19^. In particular, measuring hepatic fat deposition and liver stiffness (LS) in the same cross-sectional area is now possible with advances in echographic methods and the relationship between these parameters and blood flow velocity should be investigated.

However, no study to date comprehensively assessed variations in hepatic blood flow, LS, and liver steatosis. Given that the detection of hepatic fibrosis and lipidosis in early stages of chronic liver disease is crucial, we aimed to determine the relationship of blood flow velocity in the portal vein with LS measured using two recently introduced methods, TE with controlled attenuation parameter (CAP) and 2D-SWE with attenuation imaging (ATI)^20,21^, in patients with chronic liver disease. We investigated the effects of LS and fatty infiltration in the liver on Doppler flow hemodynamics of the portal vein.

## Results

### Patients

Twenty-eight patients, including 12 male (43%) and 16 female (57%) patients, were enrolled in the study. Table 1 summarizes the demographic profile and laboratory data of the study patients. The causes of chronic liver disease were NAFLD, chronic hepatitis C virus infection, alcoholic disease, and chronic hepatitis B virus infection in 19 (68%), 6 (21.5%), 2 (7%), and 1 (3.5%) patient, respectively. One patient with chronic hepatitis C infection had cirrhosis, whereas the only patient with chronic hepatitis B infection was a carrier; the remaining patients had chronic hepatitis with mildly abnormal liver function. In the study cohort, 25 of 28 patients (89%) were evaluated with CT, MRI, and endoscopy in the last two years of which 2 patients with compensated cirrhosis had collateral blood vessels. The remaining three patients were diagnosed with symptomatic mild liver damage, not cirrhosis, by the attending physician. Based on the clinical profile, 7 of the 28 patients (25%) were taking Ca inhibitors and angiotensin receptor blockers and 1 patient was taking a beta-blocker. The median (range) values for aspartate and alanine transaminases and platelet count were 27 (18–110) IU/L, 32 (10–195) IU/L, and 23.4 (9.2–37.4) × 10^4^/µL, respectively.

**Table 1:** Patients characteristics (*n* = 28).

Parameter	
Male sex	n = 12 (43%)
Age, years	58.0 (22-80)
BMI, kg/m^2^	24.8 (18.3–39.2)
AST, IU/L	27 (18–110)
ALT, IU/L	32 (10–195)
Platelet count, × 10^4^/µL	23.4 (9.2–37.4)
Liver disease etiology	
NAFLD	19
ALD	2
HCV	6
HBV	1
FIB-4 index	1.44 (0.35–4.81)
FibroScan (TE)	
CAP, dB/m	293.5 (150–400)
LSM, kPa	5.15 (2.9–20.4)
SWE/ATI/SWD	
ATI, dB/cm/MHz	0.80 (0.52–1.46)
2D-SWE (liver), m/s	1.41 (1.10–2.33)
2D-SWE (liver), kPa	5.90 (3.63–21.77)
2D-SWE (spleen), m/s	1.70 (0.77–4.21)
2D-SWE (spleen), kPa	9.67 (3.37–48.43)
SWD (liver), (m/s)/kHz	11.1 (7.3–25.0)
Right portal vein	
V_max_, cm/s	17.6 (11.7–36.3)
CirC, mm	24.7 (16.9–34.2)

Conversion to liver stiffness obtained by 2D-SWE is calculated by E (kPa) = ρV^2^ and displayed. ρ is the density and v is the speed of the shear wave (m/s).

Data are shown as n (%) or median and range. *BMI* body mass index, *AST* aspartate aminotransferase, *ALT* alanine aminotransferase, *AST* aspartate aminotransferase, *ALD* alcoholic liver disease, *HCV* hepatitis C virus, *HBV* hepatitis B virus, *FIB-4* Fibrosis-4, *TE* transient elastography, *CAP* controlled attenuation parameter, *LSM* liver stiffness measurement, *SWE* Shear wave elastography, *ATI* Attenuation imaging, *2D* Two-dimensional, *SWD* Shear wave dispersion, *CirC* circumference.

### Correlation between LS values obtained with TE and 2D-SWE, and steatosis values obtained with CAP and ATI

**Figure 1:**
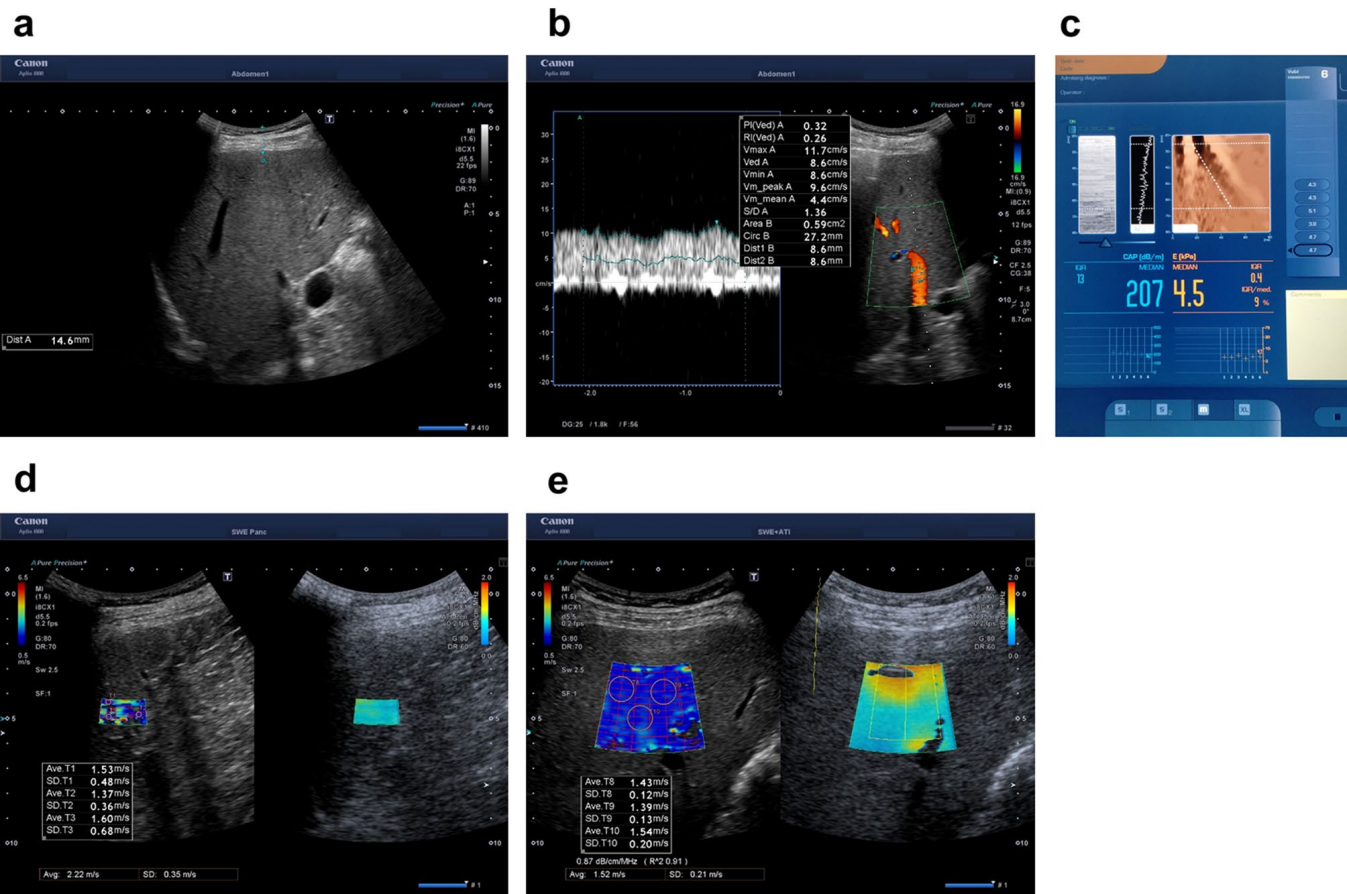
Methods used for liver evaluation. (**a**) B-mode image. (**b**) Transient elastography (TE) and controlled attenuation parameter (CAP) with FibroScan. Sample display showing the echo M-scan on the left, single-line amplitude A-scan in the middle, and the displacement M-mode after a vibration-controlled impulse push on the surface on the right. Numeric values for CAP are displayed on the left (db/m) and those for TE are displayed on the right (kPa). (**c**) Illustration of a 2D-SWE measurement and an attenuation image (ATI) acquisition of right liver lobe performed using Aplio i800. Elasticity map was displayed on the left and the level of attenuation was color-coded and displayed in the ROI on the right. Each numeric results expressed in kPa and in dB/cm/Mz are displayed on the bottom of the image. (**d**) Illustration of a 2D-SWE measurement of spleen performed using Aplio i800. (**e**) Illustration of the V_max_ measurement of right portal vein.

**Figure 2:**
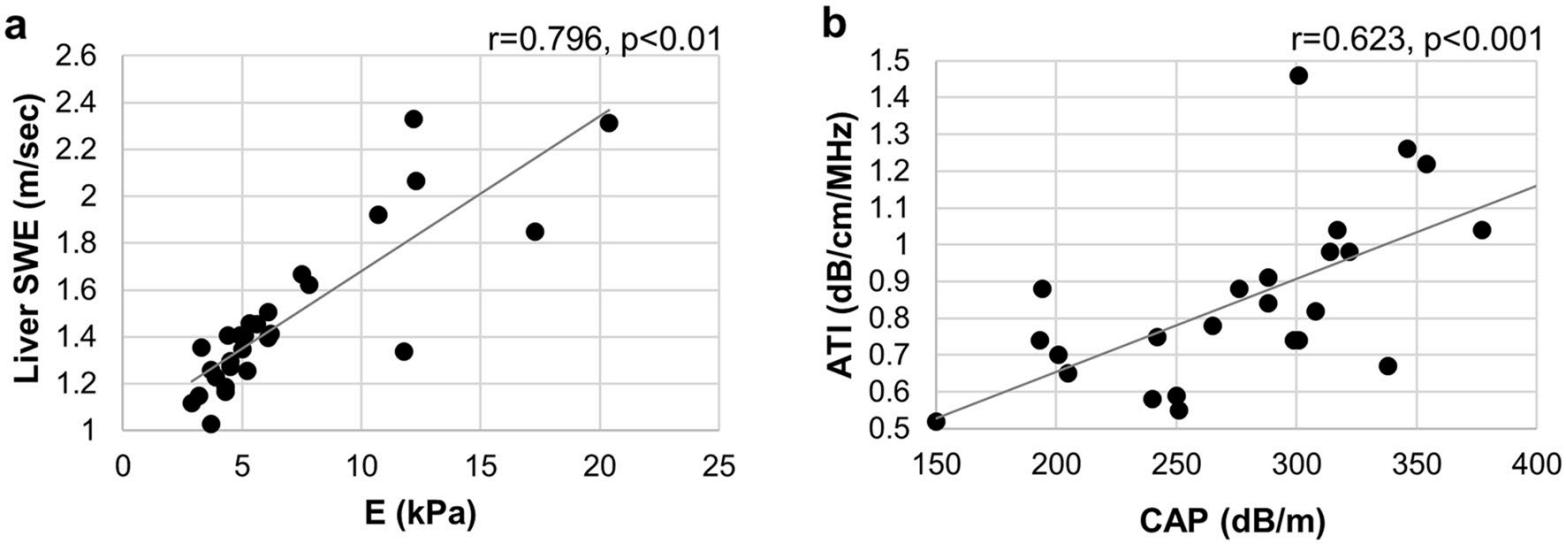
Correlation between the change in (**a**) liver stiffness obtained by 2D-SWE and by TE with Fibroscan, and in (**b**) liver steatosis by ATI and CAP.

B-mode examination, Doppler ultrasound, transient elastography by FibroScan, and 2D-SWE of spleen and liver were performed (Fig. 1a–d). The median LS values based on 2D-SWE and TE evaluation were 1.41 (1.10–2.33) m/s and 5.15 (2.9–20.4) kPa, respectively. The LS values obtained with TE and 2D-SWE exhibited a strong and significant positive correlation (r = 0.796, p < 0.01; Fig. 2a). Similarly, a strong and significant correlation was present between the steatosis values obtained with CAP and ATI (r = 0.623, p < 0.001; Fig. 2b).

### Correlation between spleen stiffness values obtained with 2D-SWE and LS values obtained with TE and 2D-SWE

**Figure 3:**
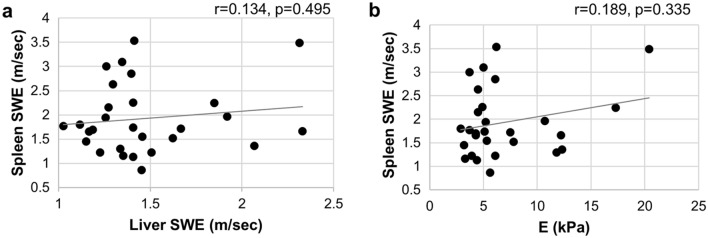
Correlation between the change in spleen stiffness obtained by SWE and liver stiffness by (**a**) 2D-SWE, and (**b**) TE with Fibroscan.

The median spleen stiffness value was 1.70 (0.77–4.21) m/sec based on 2D-SWE. However, spleen stiffness did not correlate with LS values obtained with 2D-SWE and TE (Fig. 3a,b).

### Correlation between peak velocity of the right portal vein and LS values obtained with TE and 2D-SWE

**Figure 4:**
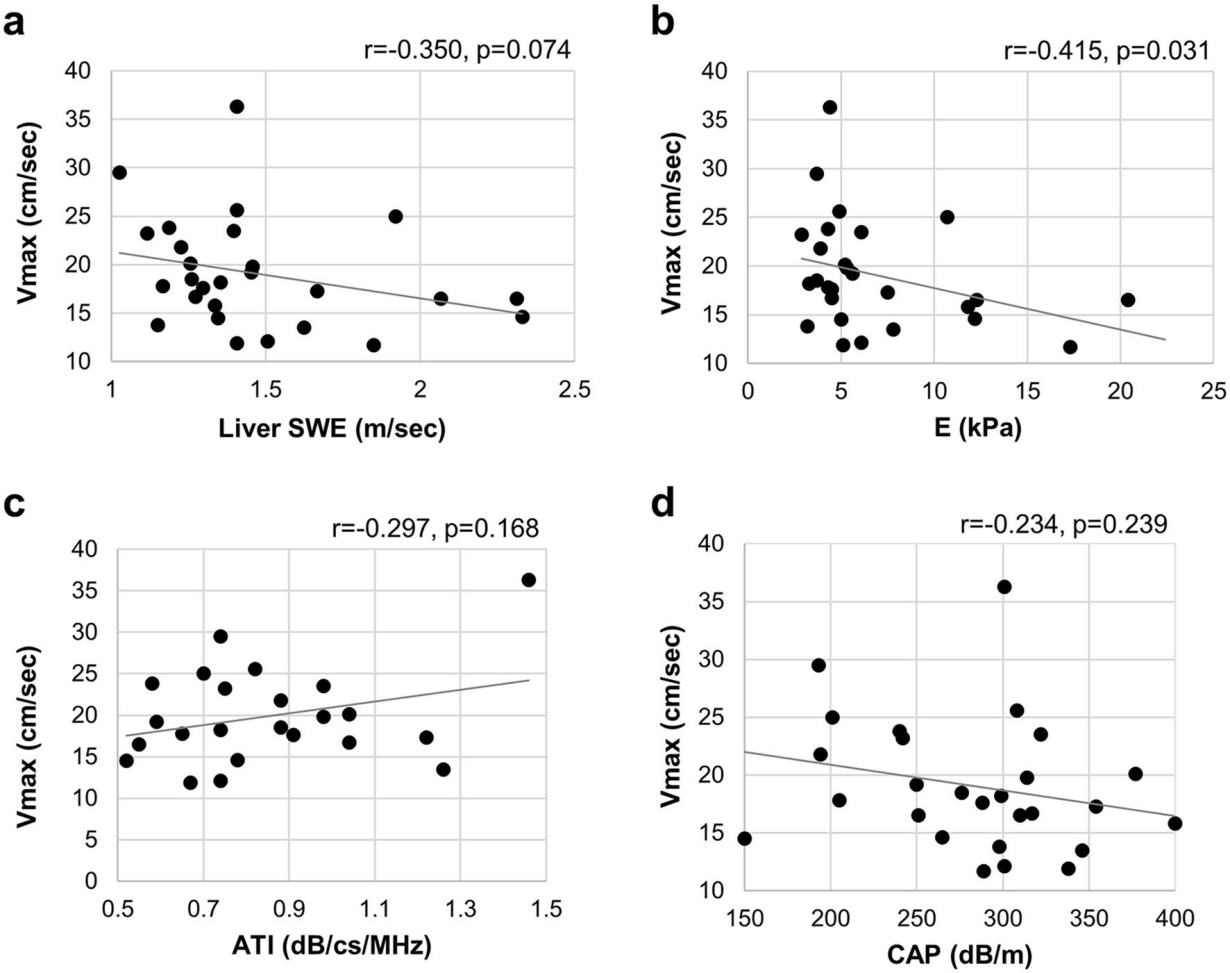
Correlation between the change in V_max_ of right portal vein and (**a**) liver stiffness by 2D-SWE, (**b**) liver stiffness by TE with Fibroscan, (**c**) liver steatosis by ATI, and (**d**) liver steatosis by CAP with Fibroscan.

The median V_max_ of the right portal vein was 17.6 (11.7–36.3) cm/s. The association between V_max_ and the LS value by 2D-SWE tended to be inversely correlated (Fig. 4a). Furthermore, a strong inverse correlation was present between the V_max_ and LS value obtained with TE (Fig. 4b). However, the V_max_ of the right portal vein was not correlated with the steatosis values obtained with CAP, ATI and shear wave dispersion (SWD) (Fig. 4c,d, and Supplementary Fig. S1a).

### Comparison of peak velocity of the right portal vein and FIB-4 index for detecting liver fibrosis

**Figure 5:**
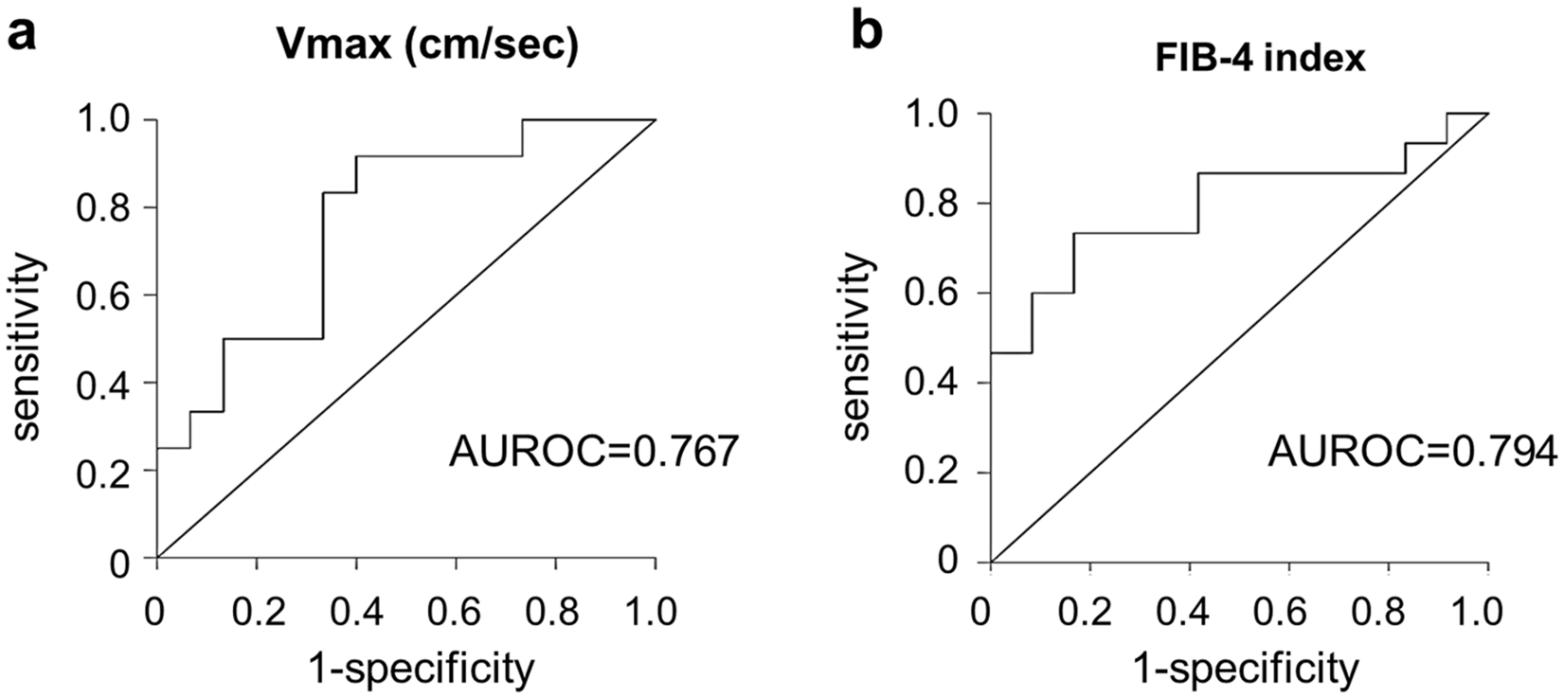
Area under the receiver-operating characteristic curves (AUROC) of (**a**) velocity of right portal vein and (**b**) Fibrosis (FIB)-4 index for predicting liver stiffness measured by TE with Fibroscan (> 5 kPa).

According to cut-off values based on 2020 SRU (Society of Radiologists in Ultrasound) Consensus Statement (vendor-neutral), LS value obtained with TE ≤ 5 kPa is normal (high probably of being normal). Although the cut-off value of LS for fibrosis stage 1 (portal fibrosis without septa) discrimination varies slightly among chronic liver diseases, in this study, LS value ≤ 5 kPa was considered normal and LS value > 5 kPa was considered as possible mild fibrosis in order not to miss early fibrosis^22^. The optimal V_max_ cutoff value for discriminating liver fibrosis with an LS value of > 5 kPa obtained with TE was < 17 cm/s (area under the receiver operating characteristic [ROC] curve [AUROC], 0.767; sensitivity, 83.3%; specificity, 60%; positive predictive value, 81.8%; negative predictive value, 66.7%; Fig. 5a). The ability of V_max_ in discriminating fibrosis (AUROC, 0.767; Fig. 5a) was comparable with that of the FIB-4 index (AUROC, 0.794; Fig. 5b), which is widely used to measure fibrosis in patients with NAFLD. If the V_max_ cutoff value for discriminating liver fibrosis with an LS value of > 5 kPa obtained with TE was set to < 20 cm/s, the specificity increased to 80% although the sensitivity decreased to 50% (data not shown).

## Discussion

Consistent with recent reports, we confirmed that LS obtained using TE and SWE, was strongly correlated with steatosis obtained using ATI and CAP in the present study^21,23^. We also found that the V_max_ of the right portal vein tended to be inversely correlated with LS based on 2D-SWE, whereas its correlation with LS based on TE was significant. These results suggest that portal blood flow velocity declines with increasing LS and worsening liver fibrosis. Early identification of the progression of fibrosis in chronic hepatitis to cirrhosis and hepatocellular carcinoma is critical, and decreased portal blood flow is a useful approach to determine increased LS, which is a simple and useful approach even in facilities where LS measurement is difficult. In patients with chronic hepatitis, a portal blood flow of < 17 cm/s could be a potential indicator of early-stage liver fibrosis, which is defined as an LS value of > 5 kPa based on TE.

In healthy individuals, the portal vein is responsible for 70–75% of all hepatic blood supply. The main portal vein enters the liver at the hilar region and branches into the right and left portal veins. Normal Doppler waveform of the portal veins demonstrates continuous antegrade flow toward the liver, with a mildly undulating pattern. Flow is affected by respiration and cardiac pulsatility, although the effect is normally mild. The mean velocity in the portal vein is typically 15–30 cm/s, while the normal V_max_ has a broad range from 15 to 40 cm/s. In general, variations in main portal vein velocity are small, with the lowest velocity measuring more than half the highest velocity^24^. Although many studies investigated the relationship between fatty liver grade and LS in patients with chronic liver disease, few recent reports examined portal vein velocity and LS. Portal vein velocity is considered to be influenced by blood viscosity, portal pressure, A–P/P–V shunt, and collateral vessel development. Despite general consensus that mean maximum portal vein velocity is decreased in patients with cirrhosis^25,26^, there are no standards for predicting liver fibrosis because portal blood flow is influenced by numerous factors, such as changes in body position, phase of respiration, timing of meals, exercise, and cardiac output. Portal flow may be unaltered due to a combination of high inflow from splanchnic organs and increased resistance within the liver. However, a recent study has reported that portal vein velocity may be useful as a noninvasive triage test for the selection of high-risk patients with cirrhosis who should be referred to and could benefit from esophagogastroduodenoscopy^27^. A portal vein velocity value of < 19 cm/s was shown to be a predictive marker for esophageal varices^27^. Our analyses showing that the velocity of the right portal vein is inversely correlated with LS in patients with relatively mild liver injury is consistent with these reports but should be carefully examined in patients with cirrhosis accompanied with collateral blood vessels, ascites, and splenomegaly. In addition, the correlation between Vmax of the right portal vein and LS values by TE was reexamined after excluding 2 patients with cirrhosis. A strong correlation was still shown between them (Supplementary Fig. S3a). Therefore, Vmax measurement may be useful even in chronic hepatitis.

Although the peak flow velocity of the portal vein is usually 15–40 cm/s, we found that a V_max_ of < 17 cm/s in the right main portal vein was a useful fibrosis predictor in patients with chronic liver injury. Importantly, the AUROC of V_max_ was comparable that of the FIB-4 index, which has demonstrated utility in the identification of patients with liver fibrosis in daily clinical practice. this index is an easy-to-use and reliable marker used for fibrosis in NAFLD. The practice guidance for NAFLD created by the American Association for the Study of Liver Diseases in 2018^28^ states that the NAFLD fibrosis score and FIB-4 index are clinically more useful noninvasive tests (NITs) in identifying patients with liver fibrosis. A recent study using a FIB-4 score of < 1.3 as a cutoff for low risk for advanced liver fibrosis (> Kleiner F3) indicated that annual assessments in primary care rather than referral to a specialist should be recommended for such patients^29^. However, the FIB-4 index increases with age, necessitating correction according to patient age^30^. In the present study, the portal vein velocity was considered to have a strong discrimination ability since age was not factor requiring correction.

Several limitations of the present study should be acknowledged. As a major limitation, biopsies were not performed to confirm the diagnosis of hepatic fibrosis or fatty liver or to assess disease severity. In addition, the study cohort size was small and lacked a wide range of patients from different age groups, which prevented comparisons with the FIB4-index, which includes an age component. Previous reports demonstrated that the pulsatility index and the mean velocity of portal blood flow decreased with increasing severity of fatty infiltration^18,19^. Therefore, extensive fatty infiltration might lead to false positivity for liver fibrosis in patients evaluated using portal vein flow. In the present study, many patients had fatty liver and a probable possibility that the portal vein velocity was decreased due to fatty hepatocytes in the absence of fibrosis. However, steatosis values obtained with CAP and ATI did not correlate with the V_max_ of right portal vein in the present study (Fig. 4c,d). Furthermore, some patients had normal LS despite a portal blood flow velocity of < 17 cm/s, whereas no patients with severe LS had a high blood flow velocity. The review of the patient records for past evaluations using CT, MRI, and endoscopy indicated that none of the patients had portal vein thrombus or hepatocellular carcinoma and two patients had collateral blood vessels. In both the patients with collateral blood vessels, the V_max_ was lower than 17 cm/s and the LS value based on TE was markedly higher than 5 kPa. Additionally, seven patients were taking calcium channel blockers and angiotensin receptor blockers, and one patient was taking beta-blockers. The V_max_ was < 17 cm/s in 4 out of 8 patients on antihypertensive medications, but none of them had a V_max_ of < 17 cm/s despite an LS value of < 5 kPa based on TE. However, further studies with a larger sample size and comparison of patients before and after antihypertensive medication are required to reveal the effect of antihypertensive medication on V_max_. We also compared V_max_, V_min_, and flow volume of the right portal vein with LS value of > 8.2 on TE, which indicates fibrosis above F2, but no correlation (p = 0.157) was found as only six patients with E > 8.2 were included in the present study. Additionally, the AUROC of V_max_ for detecting LSM > 8.2 was 0.698, which is inferior to the AUROC of FIB-4 (0.873), despite the lack of a significantly difference (data not shown). Therefore, a V_max_ of < 17 m/s with Doppler ultrasound was relatively sensitive for predicting liver fibrosis but had low specificity.

Marked hemodynamic changes in the portal vein of patients with chronic liver diseases, such as those with NAFLD, are expected to be increasingly observed as a clinical presentation in the future; these changes were also correlated with the severity of LS. During the progression of non-alcoholic steatohepatitis, liver fibrosis worsens but steatosis decreases in the so-called burn-out state^31,32^. Therefore, the establishment of cutoff values for the V_max_ of the portal vein, which reflects both liver fibrosis and steatosis, is needed in future studies. Moreover, histopathological assessment of liver biopsies remains the gold standard for the evaluation of hepatic fibrosis. We believe in the utility of liver biopsy, which is an invasive and uncomfortable procedure with limitations such as the risk of complications, sampling errors, low acceptance by patients, and inconvenience. We suggest that its utilization can be reduced by incorporating the evaluation of hemodynamic changes in the portal vein.

Needless to say, transient elastography performs best with regard to the ruling out of advanced fibrosis^33,34^. On the other hand, even F1 or F2 fibrosis stage has been reported to associate independently with long-term overall mortality and liver transplantation^35^. These results, which showed a decrease of the Vmax of the right portal vein, could be a potential indicator of early-stage liver fibrosis in patients with chronic hepatitis and should be confirmed in future studies including larger cohorts. In summary, because many facilities do not have FibroScan or do not utilize abdominal echo with SWE capability, measuring the velocity of the right main portal vein, an easily measurable parameter, should be considered as a highly useful approach to identify patients with liver fibrosis.

## Methods

### Study design, participants, and inclusion and exclusion criteria

This study was a single-arm retrospective study. A total of 28 patients already diagnosed with NAFLD, hepatitis B or C virus infection, or alcoholic liver disease that underwent abdominal ultrasound between June 1, 2021 and October 31, 2021 were enrolled in the study. Patients diagnosed with acute liver injury were excluded. A CAP value of 248 dB/m or higher on the FibroScan was diagnosed with fatty liver of any degree^36^. In the present study, the CAP values of the 19 NAFLD patients ranged from 248 to 400 dB/m, with a median value of 301 dB/m. The study did not include a control group. However, 5 out of 28 patients included in the study had achieved normalized liver function at the time of examination for the study. Most of the patients had chronic hepatitis with a background of fatty liver, followed by those with alcoholic liver disease and viral hepatitis. The other exclusion criteria were congestive liver with heart failure, primary biliary cirrhosis with bile stasis, ascites, presence of a pacemaker, and pregnancy. The present study was conducted in accordance with and fully complied with the standards of the Declaration of Helsinki. And the study protocol was approved by the local ethics committee of Kyoto Prefectural University of Medicine (approval no: ERB-C-1890) and was in accordance with the guidelines for for research involving human participants of the same institution. Written informed consent was obtained from all study the participants.

### Ultrasound, 2D-SWE/ATI/SWD, and TE measurement

Blood tests, FibroScan, 2D-SWE/ATI/SWD, and B-mode/Doppler ultrasound of the portal vein were performed at the same time with the patients fasting for more than six hours. The patients were in the supine position, with the right arm in maximum abduction, and intercostal approach was used to evaluate the right liver lobe with 5–10 s of breath-hold periods. All the examinations were performed using a 4.0-MHz convex probe with a color-coded Doppler ultrasound device (Aplio i800; Canon Medical Systems, Tochigi, Japan).

First, B-mode examination was performed and parenchymal echogenicity was evaluated in all the patients (Fig. 1a). In Doppler ultrasound, the angle between the examined vascular structure and the ultrasound beam was maintained at < 60°. The flow velocity scale was adjusted for each examination. In Doppler ultrasound, V_max_ and circumference of the right portal vein were measured (Fig. 1b).

In the same session, LS and steatosis were evaluated using TE with CAP implemented on a FibroScan 502 Touch device (Echosens, Paris, France) and 2D-SWE with ATI installed on an Aplio i800 ultrasound system. In all the patients, 10 valid LS measurements were targeted using the standard M probe (transducer frequency, 3.5 MHz) or the XL probe (transducer frequency, 2.5 MHz). The M and XL probes were used in patients with skin–liver capsule distances of < 25 mm and ≥ 25 mm, respectively, according to the manufacturer’s recommendations (Fig. 1c). The median value of the 10 valid LS measurements was calculated, and the results were expressed as kPa. Reliable measurements were defined as those with an interquartile range (IQR)/median ratio (IQR/M) of < 30%.

LS measurements with 2D-SWE were performed with a 4.0 MHz convex transducer (PVI-475BX; Canon Medical Systems). For LS measurements using 2D-SWE with ATI and SWD, the acquisition protocol proposed by the manufacturer was followed. Specifically, with the patients in supine position, the intercostal window used in SWE was used and the probe was placed perpendicular to the liver surface. A region of interest (ROI), approximately 3 × 4 cm or 2 × 1 cm in dimensions, overlaid on the B-mode image was placed in the right lobe of the liver or spleen, respectively, at the intercostal space (Fig. 1d,e). The ROI box was placed in a relatively homogeneous region of the right lobe of the liver at least 2 cm below the liver or spleen capsule, while avoiding areas of large vessels. An IQR/M of < 30% was considered to indicate SWE measurements with good precision. The median value of three measurements was determined, and reliable ATI measurements of liver steatosis were defined as those with an IQR/M of < 30% and an R2 of > 0.90, a quality parameter displayed on the screen below the greyscale image and was recommended by the manufacturer^34,37^. The system automatically calculated the median values and IQR of the valid measurements. Overall, ultrasound, 2D-SWE/ATI, and TE were performed by Sakai T. (RMS-no: 3491) and Kitano S. (no: 3672, respectively), who are Japan Society of Ultrasonics-registered medical sonographers with 23 and 25 years of ultrasonography experience, respectively.

### FIB-4 index

The FIB-4 index was calculated using the following formula: FIB-4 = age (years) × aspartate transaminase (U/L) / [platelet count (10^9^/L) × ALT1/2 (U/L)]^38^. The FIB4 index is widely used for the diagnosis of fibrosis in HCV hepatitis and HBV hepatitis^39,40^.

### Statistical analysis

All statistical analyses were performed using SPSS software v.17 (SPSS, Chicago, IL, USA). Descriptive statistics were used for clinical, anthropometric, and demographic data of the patients. The Kolmogorov–Smirnov test was used to evaluate the distribution of numerical variables. Numerical variables with normal distribution were presented as means ± standard deviation, whereas variables with non-normal distribution were presented as medians with ranges. Qualitative variables were presented as numbers and percentages. The differences between the groups were assessed using Student’s *t* test for continuous variables with normal distribution and the Mann–Whitney *U* test for continuous variables with non-normal distribution. Fisher’s and Pearson’s chi-squared tests were used to compare proportions. To determine the cutoff values of V_max_ of the right portal vein in differentiating significant liver fibrosis and steatosis, we calculated the sensitivity and specificity of each value and constructed ROC curves by plotting the sensitivity against (1 − specificity) at each value. The diagnostic performance of the velocity of right portal vain was assessed with ROC curve analysis. The most commonly used index of accuracy was AUROC, with AUROC values closer to 1.0 indicating higher diagnostic accuracy. Positive and negative predictive values were calculated. Spearman’s coefficient was used to examine correlations, and Pearson’s coefficient of precision was used to compare LS using TE with CAP and 2D-SWE with ATI. Additionally, 95% confidence intervals were calculated for each predictive test and a p value of < 0.05 was considered to indicate statistical significance for all the tests.

### Ethical statement

This study was approved by the local ethics committee of Kyoto Prefectural University of Medicine (the approval number: ERB-C-1890). Written informed consent was obtained from all study participants.

## Supplementary Information

Supplementary Information.

## Data Availability

The datasets generated and/or analyzed during the current study are not publicly available due to ethical restrictions imposed by an IRB but are available from the corresponding author on reasonable request.

## Abbreviations

LS: Liver stiffness
TE: Transient elastography
CAP: Controlled attenuation parameter
2D-SWE: Two-dimensional shear-wave elastography
ATI: Attenuation imaging
NAFLD: Nonalcoholic fatty liver disease
SWD: Shear wave dispersion
AUROC: Area under receiver operating characteristic curve
