# Supplementary material for: Comparison of portal vein hemodynamics with ultrasound-based elastography for the prediction of liver fibrosis in patients with chronic liver disease
Source: Sci Rep. 2023 Feb 28;13:3425. doi: 10.1038/s41598-023-30279-7 (PMC9975193; doi:10.1038/s41598-023-30279-7)
Supplement: Supplementary file 1 — Supplementary Information. [file 41598_2023_30279_MOESM1_ESM.pdf]

SUPPLEMENTARY　INFORMATION

Comparison of portal vein hemodynamics with ultrasound-based elastography for the prediction of liver fibrosis in patients with chronic liver disease

Kanji Yamaguchi[1], Yuya Seko[1], Takamitsu Sakai[2], Satomi Kitano[2], Hiromi Okabe[2], Seita Kataoka[1], Michihisa Moriguchi[1], Atsushi Umemura[3], and Yoshito Itoh[1]

Contents:

・Supplementary Figures and Legends.

a

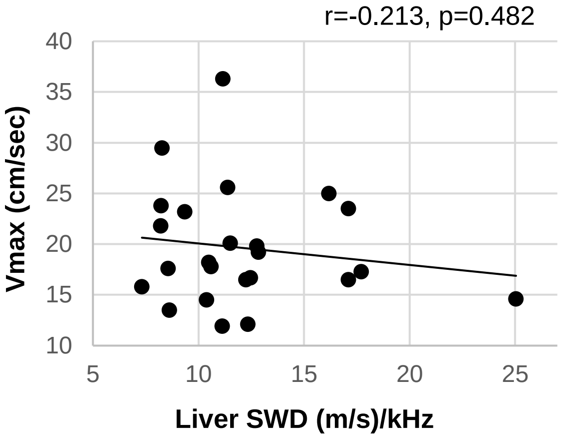

Supplementary Figure S1. (a) Correlation between the change in V_{max} of right portal vein and liver viscosity score by SWD.

Supplementary Figure S2

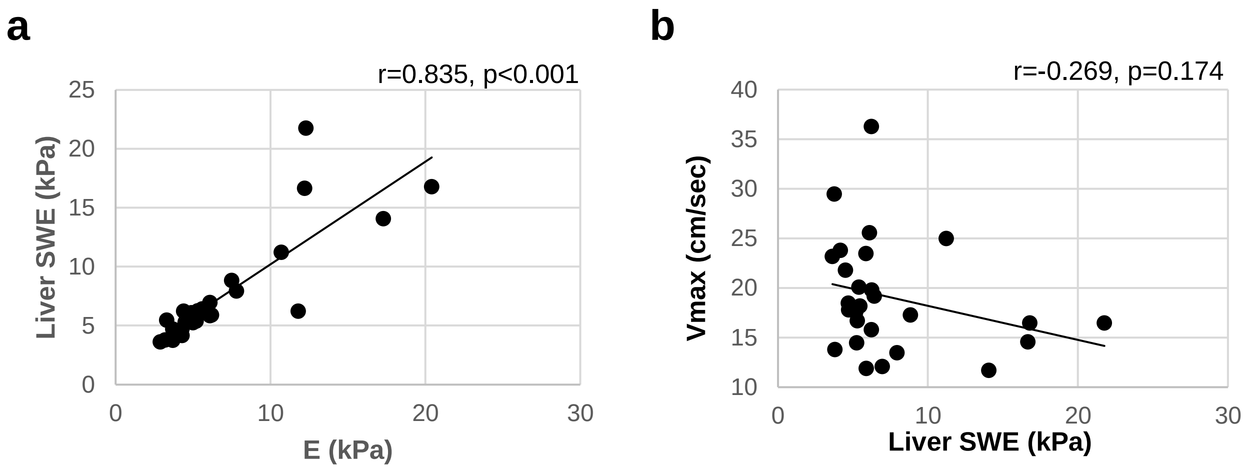

Supplementary Figure S2. Correlation between the change in liver stiffness obtained by 2D-SWE (kPa) and by TE with Fibroscan (a), and Vmax of right portal vein (b). Conversion to liver stiffness obtained by 2D-SWE is calculated by E (kPa) $= 3\rho V^2$ and displayed. ρ is the density and v is the speed of the shear wave (m/sec).

a

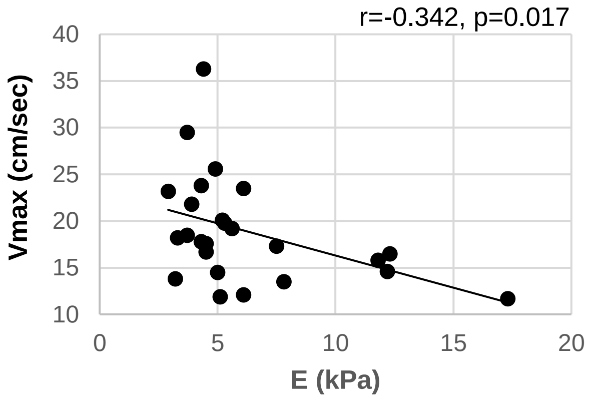

Supplementary Figure S3. (a) Correlation between the change in V_{max} of right portal vein and liver stiffness obtained by TE with Fibroscan without two LC patients.
